# Sex-Specific Associations of Childhood BMI Patterns with Cardiometabolic Risk: An 11-Year Korean Longitudinal Study

**DOI:** 10.3390/children12070821

**Published:** 2025-06-21

**Authors:** Hyo-Jin Kim, Sarang Jeong, Joo Hyun Lim, Dankyu Yoon

**Affiliations:** 1 Division of Endocrine and Kidney Disease Research, Department of Chronic Disease Convergence Research, National Institute of Health, Cheongju 28159, Republic of Korea; 2 The Korean Institute of Nutrition, Hallym University, Chuncheon 24252, Republic of Korea

**Keywords:** body mass index, childhood obesity, cardiometabolic risk, longitudinal study

## Abstract

**Background/Objectives:** Childhood overweight/obesity status is a critical risk factor for adverse cardiometabolic outcomes. We aimed to evaluate the sex-specific associations between a maintained childhood overweight status and late-adolescent cardiometabolic risk factors using data from a Korean longitudinal study. **Methods**: We used data from the Korean Children-Adolescents Study, a prospective cohort of children enrolled at age 7 and followed annually from 2005 to 2020. Among participants who were followed at least once, a total of 899 children (438 boys, 461 girls) with consistent body mass index (BMI) status at ages 7–9 and 10–12 were included in the analysis. Participants were categorized into two groups on the basis of BMI: normal weight maintenance and overweight maintenance. Multivariable linear regression was used to examine the associations between BMI patterns and cardiometabolic risk factors, with adjustments for covariates. **Results**: Among the 899 children (mean age: 7.1 ± 0.4 years, 48.7% boys), 12.8% of boys and 5.9% of girls were classified into the overweight maintenance group. Boys in the overweight maintenance group had significantly greater BMIs, waist circumferences (WC), body fat percentages, trunk fat mass, and aspartate aminotransferase and alanine aminotransferase levels at ages 15 and 18. Girls in the same group had elevated BMI, WC, body fat percentage, trunk fat mass, and blood pressure and experienced earlier pubertal onset. **Conclusions**: Maintaining an overweight status during childhood is associated with adverse cardiometabolic profiles in adolescence, with sex-specific differences. These findings highlight the importance of early, sex-specific interventions to prevent long-term health risks associated with childhood obesity.

## 1. Introduction

The global prevalence of childhood overweight and obesity has increased continuously since 1990 [1]. Over the past two decades, 1 in 5 children and adolescents had an overweight status, and the prevalence of obesity from 2012 to 2023 increased 1.5 times compared with that from 2000 to 2011 [2]. Childhood obesity is more likely to continue into adulthood [3] and is associated with an increased risk of developing both cardiometabolic diseases [4,5] and their major risk factors—such as dyslipidemia, hypertension, and insulin resistance (IR)—which are already present in children and adolescents with obesity [6]. Therefore, understanding the effects of childhood overweight and obesity status on adult health, particularly on the risk of developing cardiometabolic diseases in adulthood, is important.

Most epidemiological studies have used body mass index (BMI) at a single time point to assess childhood obesity [5,7]. However, childhood and adolescence are dynamic periods characterized by rapid physical and hormonal changes, including sex-specific differences in body composition. Therefore, classifications based on BMI measured at a single time point may be insufficient to capture meaningful weight development patterns throughout growth. To address this, an increasing number of studies have adopted trajectory-based approaches, which analyze how BMI changes over time rather than relying on a single measurement [8]. BMI trajectories refer to longitudinal patterns of weight status assessed across multiple time points. These approaches allow researchers to evaluate whether an individual maintains, gains, or loses weight over time, thereby providing a more dynamic and informative picture of long-term health risks. Recent studies have begun to examine BMI trajectories using multiple time-point assessments [8,9,10] and have reported associations between a persistent overweight status and increased risk of experiencing cardiometabolic outcomes [9,10].

However, most of these studies have been conducted in Western populations. Findings from these populations may not be fully generalizable to East Asian contexts due to differences in genetic background, body composition, dietary patterns, and lifestyle behaviors. Previous studies [11,12] have shown that Asian populations tend to have higher body fat percentages at lower BMIs than their Western counterparts, which may partly explain the elevated metabolic risk observed even at relatively low BMI levels. While most of this evidence has been reported in adults, similar trends have been observed in children. According to Liu et al. [13], Asian children tend to have higher body fat than Caucasian peers at the same BMI levels, which may result in an underestimation of obesity-related health risks when using standard BMI classifications. These findings highlight the need for population-specific research focusing on Asian children, including in Korean populations.

To our knowledge, few longitudinal studies have examined how persistent overweight status in childhood relates to cardiometabolic risk in adolescence, particularly in East Asian populations. Moreover, sex-specific differences in these associations remain understudied. Therefore, in the present study, we used annual anthropometric measurements from a cohort of Korean children and adolescents to identify BMI patterns during childhood. The aim of this study was to investigate the sex-specific associations between these BMI patterns and cardiometabolic risk in late adolescence.

## 2. Materials and Methods

### 2.1. Study Population

**Figure 1:**
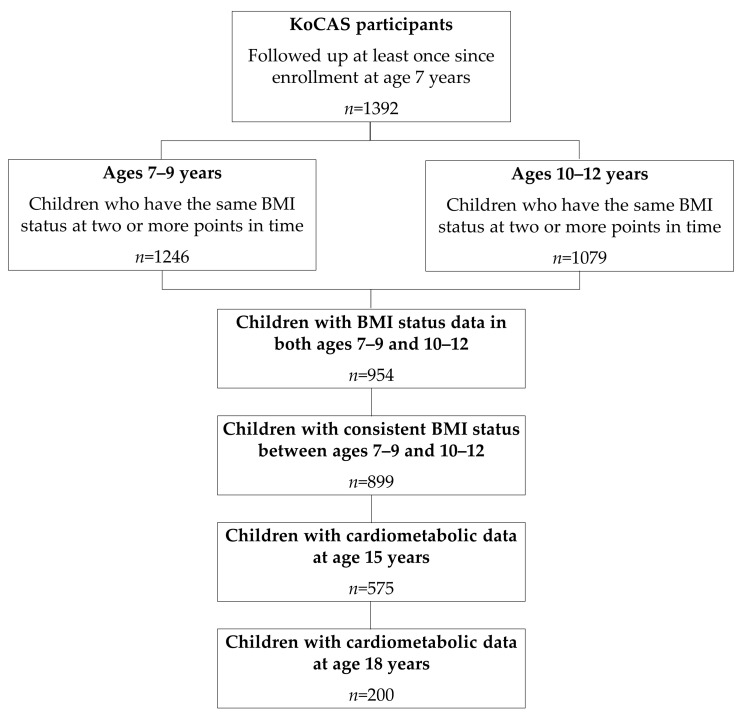
Participant flowchart. Abbreviations: KoCAS, Korean Children-Adolescents Study; BMI, body mass index.

For the present study, we used data from the Korean Children-Adolescents Study (KoCAS) conducted by the National Institute of Health (NIH) [14]. To comprehensively investigate the growth patterns of Korean children, an open cohort design was employed, enrolling participants aged 7 years. Annual follow-up assessments were conducted from 2005 to 2020 (excluding 2015) in selected regions of Seoul and Gyeonggi-do, Korea. Participants were recruited from first-grade classes in elementary schools (age 7) in these regions as part of a school-based prospective study. All students were invited to participate, and the final study sample included only those who voluntarily agreed to take part in the study. A total of 1392 children were followed at least once after enrollment at age 7. Among them, 954 children had complete BMI data across two periods: ages 7–9 years and 10–12 years. Of these, 899 children who maintained the same BMI classification (normal or overweight) across both periods were included in the final analysis. Participants whose classification differed between the two periods were considered to have an inconsistent BMI status and were excluded, as defined in Section 2.5. Among them, 575 children (63.96%) had cardiometabolic measurements at age 15, and 200 children (22.25%) had cardiometabolic measurements at age 18 (Figure 1). The study was approved by the Institutional Review Board of the Korea Disease Control and Prevention Agency. Informed consent was obtained from all participating children and their parents.

### 2.2. Anthropometric Measurements

Trained personnel measured anthropometric indices using standardized protocols. Height was measured with an automatic stadiometer (DS-102; Jenix, Seoul, Republic of Korea). Weight and body composition (e.g., body fat percentage, fat-free mass, and trunk fat mass) were determined via bioimpedance analysis (BIA) (BC-418; Tanita, Tokyo, Japan). BMI was calculated as weight in kilograms divided by height in meters squared (kg/m^2^). Age- and sex-specific BMI percentiles and BMI *z* scores were calculated using the 2017 Korean National Growth Charts for Children and Adolescents [15]. Waist circumference (WC) was measured at the midpoint between the lower border of the ribcage and the iliac crest via a nonelastic tape measure. Blood pressure (BP) was measured twice with the participants in a seated position following a period of rest of at least 5 min using a mercury sphygmomanometer on the right arm; the two measurements were then averaged.

### 2.3. Biochemical Assessments

Blood samples were collected from the brachial vein after a 12 h overnight fast. The levels of triglycerides (TGs), total cholesterol (TC), high-density lipoprotein cholesterol (HDL-C), alanine aminotransferase (ALT), aspartate aminotransferase (AST), and glucose were measured using an autoanalyzer (model 7600 II; Hitachi, Tokyo, Japan). Low-density lipoprotein cholesterol (LDL-C) levels were calculated using the Friedewald formula: LDL-C = TC − [HDL-C + (TG/5)] [16]. Fasting serum insulin was measured using an enhanced chemiluminescence immunoassay analyzer (E170; Roche Diagnostics, Mannheim, Germany).

### 2.4. IR Indices

IR indices were calculated using the homeostasis model assessment of insulin resistance (HOMA-IR) index and the TG-glucose (TyG) index. The HOMA-IR index was calculated using the following formula: fasting glucose (mg/dL) × fasting insulin (μIU/mL)/405 [17]. The TyG index was calculated as Ln [fasting glucose (mg/dL) × fasting TGs (mg/dL)/2] [18].

### 2.5. BMI Classification and Pattern Grouping

We defined overweight as BMI ≥ 85th percentile and normal weight as BMI < 85th percentile, on the basis of age- and sex-specific Korean growth charts [15]. For each 3-year period (ages 7–9 and 10–12), children were classified as “overweight” if they met the overweight criteria on at least two out of three measurements, and as “normal weight” if they met the normal weight criteria on at least two out of three measurements.

Based on their weight status across both periods, participants were categorized into two BMI pattern groups: (1) normal weight maintenance group, which included children who consistently met the normal weight criteria in both periods; and (2) overweight maintenance group, which included those who consistently met the overweight criteria in both periods.

Children with inconsistent BMI classifications across the two periods (e.g., from normal weight to overweight or from overweight to normal weight) were excluded from pattern grouping.

### 2.6. Additional Variables

Information on birth weight, pubertal development, maternal demographic characteristics, parental height and weight, and physical activity (daily sedentary time and walking time) was obtained from children and parents using a self-report questionnaire once per year. Birth weight was recorded to the nearest 0.01 kg. Pubertal development was assessed using the Tanner stages, which indicate sexual development and growth in five stages for breasts, genitals, and pubic hair, as well as body changes [19]. In this study, age at onset of puberty was defined as the time of first reported Tanner stage 2 or above. Maternal education level was categorized into three groups: high school or lower, university (including junior college) or higher, and missing. Maternal employment status was categorized into three groups: not working, working, or missing. Parental BMI status was categorized into three groups—normal weight (<23 kg/m^2^), overweight (≥23 kg/m^2^), and missing—based on the Asia-Pacific criteria of the World Health Organization and the Korean Society for the Study of Obesity. Daily sedentary time was calculated as the average duration of screen viewing (TV or computer use) and reading across weekdays and weekends, and was categorized into three groups: <1 h/day, ≥1 h/day, and missing. Daily walking time was based on average walking duration per weekday and was similarly categorized into three groups: <1 h/day, ≥1 h/day, and missing. Participants with missing responses were grouped together as a separate category and included in the analysis.

### 2.7. Statistical Analysis

All analyses were performed using SAS version 9.4 (SAS Institute, Cary, NC, USA). Skewed variables were log-transformed, and normality was assessed using skewness and kurtosis. To more accurately reflect the distribution of continuous variables, both mean ± standard deviation and median (Q1, Q3) were reported. Between-group comparisons were performed via the chi-square test for categorical variables and Student’s *t* test for continuous variables. Linear regression models were used to examine associations between BMI patterns and cardiometabolic risk factors at ages 15 and 18. Both unadjusted and adjusted models were fitted. The adjusted models included baseline age, maternal education, maternal BMI status, paternal BMI status, and daily sedentary time as covariates. All analyses were stratified by sex.

To evaluate potential bias due to loss to follow-up, baseline characteristics were compared between participants who remained in follow-up and those lost to follow-up at ages 15 and 18, stratified by sex. The results are presented in Supplementary Tables S1 and S2.

For all tests, a *p* < 0.05 was considered to indicate statistical significance.

## 3. Results

### 3.1. Baseline Characteristics

In total, 899 children (438 boys, 461 girls) were included, with a mean baseline age of 7.1 years. The distribution of BMI patterns was as follows: 382 (87.2%) boys and 434 (94.1%) girls in the normal weight maintenance group and 56 (12.8%) boys and 27 (5.9%) girls in the overweight maintenance group (Table 1). The baseline characteristics by BMI pattern are shown in Table 1. Birth weight did not significantly differ between BMI groups. Boys in the overweight maintenance group were more likely than those in the normal weight maintenance group to have mothers with lower educational attainment, have overweight parents, and exhibit more sedentary behavior. Girls who maintained overweight had fathers with higher BMIs and earlier onset of puberty (9.2 ± 0.9 vs. 10.2 ± 1.1 years, *p* < 0.0001). The mean anthropometric measurements significantly differed between BMI pattern groups in both boys and girls. Children in the overweight maintenance group had significantly greater heights, BMIs, BMI *z* scores, and WCs. Boys in the overweight maintenance group had elevated diastolic BP (DBP); ALT, TC, and LDL-C levels; and TyG indices. Girls in the overweight maintenance group presented increased systolic BP (SBP) and DBP; AST, ALT, TG, and HDL-C levels; and TyG indices.

### 3.2. Cardiometabolic Profiles at Age 15

Mean cardiometabolic profiles at age 15 are presented by BMI pattern (Table 2). In boys, all cardiometabolic risk factors except height differed significantly between BMI pattern groups. Compared with boys who maintained a normal weight, those in the overweight maintenance group had higher levels of cardiometabolic risk factors. In girls, anthropometric measurements (excluding height) and SBP at age 15 were significantly greater in the overweight maintenance group than in the normal weight maintenance group. Although laboratory markers and insulin resistance indices were higher in the overweight maintenance group than in the control group, these differences were not statistically significant.

### 3.3. Cardiometabolic Profiles at Age 18

Mean cardiometabolic profiles at age 18 are presented by BMI pattern (Table 3). At age 18, height was significantly lower in the overweight maintenance group than in the normal weight group (170.8 ± 6.2 vs. 175.0 ± 5.2 cm, *p* = 0.0075), but only in boys. Other anthropometric measurements (excluding fat-free mass) were significantly greater in the overweight maintenance group than in the normal weight maintenance group for both boys and girls. Among girls, SBP and DBP were significantly elevated in the overweight maintenance group, whereas no such difference was observed in boys. Compared with those in the normal weight maintenance group, the AST and ALT levels were significantly greater in both boys and girls who maintained an overweight status. Insulin and glucose levels were significantly elevated only among girls in the overweight maintenance group.

### 3.4. Associations Between BMI Pattern and Cardiometabolic Risk Factors

Table 4 presents the associations between BMI patterns and cardiometabolic risk at ages 15 and 18 in boys. After adjustment for covariates (baseline age, maternal education, maternal BMI status, paternal BMI status, and daily sedentary time), boys in the overweight maintenance group had a significantly higher BMI (*β* = 0.254; 95% CI, 0.209 to 0.299), WC (*β* = 0.172; 95% CI, 0.138 to 0.207), body fat percentage (*β* = 0.605; 95% CI, 0.465 to 0.746), fat-free mass (*β* = 0.083; 95% CI, 0.047 to 0.120), trunk fat mass (*β* = 0.866; 95% CI, 0.551 to 1.181), SBP (*β* = 0.061; 95% CI, 0.019 to 0.102), DBP (*β* = 0.061; 95% CI, 0.014 to 0.109), AST (*β* = 0.294; 95% CI, 0.140 to 0.448), ALT (*β* = 0.761; 95% CI, 0.528 to 0.994), TG (*β* = 0.293; 95% CI, 0.002 to 0.584) and HOMA–IR index (*β* = 0.691; 95% CI, 0.343 to 1.039), as well as lower HDL-C (*β* = −0.160; 95% CI, −0.264 to −0.057) at age 15, than did those in the normal weight maintenance group. At age 18, these boys continued to have a significantly elevated BMI, WC, body fat percentage, trunk fat mass, and AST and ALT values relative to those in the normal weight group. Given the limited sample size in the overweight maintenance group at this age, these findings may have reduced statistical power and should be interpreted with caution. In girls, after adjustment for covariates, the overweight maintenance group had a higher BMI, WC, body fat percentage, fat-free mass, trunk fat mass, BP, and ALT levels, as well as lower HDL-C at age 15 and higher BMI, WC, body fat mass, trunk fat mass, BP, AST, and ALT levels at age 18 than the normal weight maintenance group did. However, due to the small number of girls in the overweight maintenance group—particularly at age 18 (*n* = 2)—these results are not presented in detail.

## 4. Discussion

Childhood overweight and obesity are recognized as important risk factors for developing cardiometabolic diseases in later life. This study aimed to investigate the sex-specific associations between childhood BMI patterns and cardiometabolic risk in late adolescence using data from a Korean longitudinal study. We found that maintaining an overweight status during childhood was associated with adverse cardiometabolic profiles in late adolescence, with age- and sex-specific variations. Boys presented with elevated liver enzyme levels and shorter mean height (at age 18), while among girls, higher blood pressure and earlier pubertal onset were observed. These results reinforce the need for early, sex-specific preventive strategies to lower the future health risks associated with maintained overweight during childhood.

Our findings are consistent with previous evidence [9,10,20] showing that childhood obesity often persists into adulthood and contributes to an increased risk of developing cardiometabolic disease. Prior longitudinal studies and meta-analyses have reported consistent associations between a persistent overweight status and elevated risks of developing hypertension, type 2 diabetes, dyslipidemia, and cardiovascular disease [9,10,20]. Similarly, our study revealed that maintaining an overweight status during childhood was associated with adverse cardiometabolic profiles in both boys and girls. Compared with their peers who maintained normal weight during childhood, boys who maintained an overweight status during childhood had significantly greater BMIs, WCs, body fat percentages, trunk fat mass, and AST and ALT levels at ages 15, and continued to exhibit elevated levels of these markers at age 18, whereas girls in the same group had higher BMIs, body fat percentage, trunk fat mass, BPs, and ALT levels at both time points.

The relationship between childhood BMI patterns and adolescent BP in our study appeared to differ by sex. While several studies have confirmed an overall association between childhood obesity and adult hypertension [9], many studies lack sex-specific analyses. A recent longitudinal study [21] revealed that males with a high increasing BMI trajectory group were at greater risk of developing hypertension, with a similar but nonsignificant trend in females. The effect was more pronounced among those with unhealthy lifestyle behaviors, such as smoking, alcohol consumption, and physical inactivity—especially in males. In our study, both boys and girls in the overweight maintenance group showed higher blood pressure levels at age 15. At age 18, this pattern was observed only among girls. This sex-specific difference may reflect differential physiological responses to sustained adiposity, although the mechanisms remain unclear [22]. By contrast, at age 18, no significant association between BMI pattern and blood pressure was observed in boys, differing from previous findings [21]. This discrepancy may be partly explained by differences in outcome measurement; the prior study assessed incident hypertension in adulthood, whereas our study examined blood pressure levels during adolescence. As such, risk-enhancing behaviors such as smoking and alcohol use—more prevalent in young adulthood—may have contributed more strongly to hypertension risk in the previous study. Additionally, unmeasured confounders, such as dietary intake or other lifestyle factors, may have influenced the observed associations in our study.

In addition, this study revealed that maintaining an overweight status during childhood was associated with elevated levels of liver enzymes, particularly AST and ALT, at ages 15 and 18 in both boys and girls. These enzymes serve as surrogate markers for liver injury and are commonly used in the screening of pediatric nonalcoholic fatty liver disease (NAFLD) or, more recently, metabolic dysfunction-associated steatotic liver disease (MASLD) [23,24,25,26]. Childhood obesity is recognized as a key contributor to hepatic steatosis and liver dysfunction [27,28]. Notably, boys in the overweight maintenance group consistently exhibited higher AST and ALT levels compared to the normal weight group at both time points. These associations remained robust after adjusting for key confounding factors, suggesting a stable relationship between having a sustained overweight status and liver enzyme elevation in boys. Although girls in the overweight group also showed elevated enzyme levels, the differences were less pronounced. This sex difference may be explained by physiological variations in adipose tissue function and distribution. Males tend to accumulate more visceral fat, whereas females store more fat subcutaneously prior to menopause [29]. Given that visceral fat is closely associated with NAFLD [30], this pattern of fat distribution may contribute to the more persistent liver enzyme elevations observed in boys.

This study also confirmed prior findings [31,32] on the link between obesity and earlier pubertal onset, particularly in girls. Pubertal development typically begins earlier in girls (8–13 years) than in boys (9–14 years) [33], and the association between obesity and earlier puberty is well established in girls [31,32]. Several studies have reported that obese girls tend to experience earlier menarche and pubertal onset than their normal-weight peers do [32]. Consistent with these findings, our study revealed significantly earlier pubertal onset only among girls in the overweight maintenance group. In contrast, the evidence for boys is less consistent [32]. While some studies have suggested a potential link between increased adiposity and earlier pubertal timing, others—such as a longitudinal study by Lee et al. [34]—have shown that a higher BMI in early childhood may actually be associated with delayed pubertal onset. A recent review also concluded that, unlike in girls, the association between obesity and pubertal timing in boys remains inconclusive [31]. Our findings reflect this uncertainty, as we did not observe a clear association between overweight status and pubertal timing in boys. We did, however, observe a sex-specific association between BMI pattern and height. Previous studies have suggested that chronic excess adiposity may promote early linear growth and skeletal maturation during childhood, which can ultimately limit final height by reducing the intensity of the pubertal growth spurt [35]. Although these mechanisms have been primarily proposed in general pediatric populations, they may partly explain the shorter height observed in overweight boys in our study.

Our study has several strengths. First, height and weight were objectively measured annually from childhood through adolescence, minimizing the risk of reporting bias. Second, the longitudinal nature of the data, which were collected over a relatively extended follow-up period, enhances the reliability of the derived BMI change patterns. Third, cardiometabolic risk factors were assessed at ages 15 and 18 rather than at a single time point, which enabled a more comprehensive understanding of the associations between BMI trajectories and cardiometabolic risk. Finally, the study provided sex-specific findings that are important for understanding the differential impact of BMI changes on cardiometabolic risk in boys and girls.

However, the study also has several limitations. First, BMI is widely used to classify overweight and obesity, but it does not differentiate between fat and lean mass, which may lead to misclassification of adiposity in children [36]. Although we included BIA-derived measures (e.g., body fat percentage, fat-free mass, and trunk fat mass) to assess body composition, BIA has limited accuracy in pediatric populations due to its sensitivity to hydration status, developmental variability, and body proportions [37]. Second, the relatively small sample size—particularly in the overweight maintenance group—may have constrained our ability to fully explore the associations between BMI change patterns and cardiometabolic risk. Furthermore, the possibility of bias due to loss to follow-up should be considered when interpreting the results. Third, participants were drawn only from specific regions in Korea, which may limit the generalizability of the findings to the broader population of Korean children. Moreover, sociocultural and institutional contexts in Korea may limit the applicability of these results to other countries. Fourth, several variables were based on self-reported questionnaires (e.g., parental BMI, maternal education, physical activity), which may be prone to recall bias or social desirability bias. Other potential confounders that were not fully considered may also have influenced the results. Multiple *p*-values were reported across descriptive and multivariable analyses, which may increase the potential for Type I error. Finally, although cardiometabolic risk factors were assessed at ages 15 and 18, additional follow-up into adulthood would have provided a more comprehensive understanding of the long-term health consequences of childhood BMI changes. To address these limitations, future studies should include mechanistic investigations to elucidate underlying pathways, randomized controlled trials with sex-stratified analyses to determine intervention efficacy across subgroups, and prospective cohort studies with standardized epidemiological measures and extended follow-up periods to establish temporal relationships. Nonetheless, conducting repeated and objective measurements in the same participants over an 11-year period constitutes a considerable methodological strength. Moreover, this study offers valuable insights into BMI change patterns over a six-year span, providing important temporal information for evaluating the relationship between obesity changes and cardiometabolic risk in children and adolescents.

Since childhood overweight is a well-established risk factor for adverse health outcomes later in life, our findings support the need for early identification and intervention to reduce the long-term burden of obesity-related conditions such as hypertension, diabetes, and cardiovascular disease. In addition, they may serve as a foundational resource for developing appropriate prevention strategies in school or community-based settings.

## 5. Conclusions

This study demonstrated that maintaining an overweight status during childhood was associated with adverse cardiometabolic outcomes in adolescence, with some sex-specific differences observed. These findings clarify how childhood BMI changes affect cardiometabolic risk by sex and may inform the development of tailored prevention strategies that consider the timing of intervention. In addition, they may serve as a foundation for effective interventions and public health policies aimed at addressing childhood obesity. Taken together, our study provides a valuable longitudinal perspective by linking objectively measured BMI changes in childhood with cardiometabolic risks at multiple adolescent stages, while accounting for sex-specific differences. Its design may contribute to a more nuanced understanding of how early weight patterns shape health risks across critical developmental periods.

## Figures and Tables

**Table 1:** Baseline characteristics of 899 children by BMI pattern.

	Boys				Girls			
	*n*	Normal Weight Maintenance Group	Overweight Maintenance Group	*p*	*n*	Normal Weight Maintenance Group	Overweight Maintenance Group	*p*
*n* ** (%)**	438	382 (87.2)	56 (12.8)		461	434 (94.1)	27 (5.9)	
**Age (years)**	438	7.1 ± 0.4	7.1 ± 0.3	0.2358	461	7.1 ± 0.4	7.2 ± 0.4	0.1334
		7.1 (6.9, 7.4)	7.2 (6.9, 7.3)			7.1 (6.8, 7.4)	7.3 (7.0, 7.5)	
**Birth weight (kg)**	349	3.3 ± 0.4	3.4 ± 0.5	0.3206	379	3.2 ± 0.4	3.3 ± 0.6	0.4843
		3.3 (3.0, 3.5)	3.3 (3.0, 3.6)			3.2 (3.0, 3.5)	3.2 (3.0, 3.5)	
**Pubertal onset age (years)**	325	12.4 ± 1.3	12.1 ± 1.6	0.3867	359	10.2 ± 1.1	9.2 ± 0.9	**<0.0001**
		12.3 (11.7, 13.0)	12.1 (11.6, 13.0)			10.1 (9.6, 10.8)	9.3 (8.3, 10.0)	
**Maternal education, ** ***n*** ** (%)**	438			**0.0149**	461			0.4015
High school or lower		68 (17.8)	19 (33.9)			96 (22.1)	9 (33.3)	
University ^1^ or higher		211 (55.2)	27 (48.2)			229 (52.8)	12 (44.5)	
Missing		103 (27.0)	10 (17.9)			109 (25.1)	6 (22.2)	
**Maternal employment status, ** ***n*** ** (%)**	438			0.7357	461			0.9401
Not working		144 (37.7)	21 (37.5)			196 (45.2)	13 (48.2)	
Working		126 (33.0)	21 (37.5)			125 (28.8)	7 (25.9)	
Missing		112 (29.3)	14 (25.0)			113 (26.0)	7 (25.9)	
**Maternal BMI status, ** ***n*** ** (%)**	438			**0.0009**	461			0.1247
Normal weight		226 (59.2)	27 (48.2)			245 (56.5)	11 (40.8)	
Overweight		45 (11.8)	17 (30.4)			68 (15.7)	8 (29.6)	
Missing		111 (29.1)	12 (21.4)			121 (27.9)	8 (29.6)	
**Paternal BMI status, ** ***n*** ** (%)**	438			**0.0307**	461			0.2551
Normal weight		125 (32.7)	13 (23.2)			136 (31.3)	5 (18.5)	
Overweight		141 (36.9)	31 (55.4)			177 (40.8)	15 (55.6)	
Missing		116 (30.4)	12 (21.4)			121 (27.9)	7 (25.9)	
**Sedentary time, ** ***n*** ** (%)**	438			**0.0388**	461			0.1524
<1 h/day		91 (23.8)	12 (21.4)			80 (18.4)	9 (33.3)	
≥1 h/day		108 (28.3)	25 (44.6)			155 (35.7)	7 (25.9)	
Missing		183 (47.9)	19 (33.9)			199 (45.9)	11 (40.8)	
**Walking time, ** ***n*** ** (%)**	438			0.5941	461			0.4385
<1 h/day		204 (53.4)	32 (57.1)			243 (56.0)	13 (48.2)	
≥1 h/day		79 (20.7)	13 (23.2)			98 (22.6)	9 (33.3)	
Missing		99 (25.9)	11 (19.6)			93 (21.4)	5 (18.5)	
**Anthropometric measurements**								
Height (cm)	438	123.0 ± 5.2	125.3 ± 5.3	**0.0027**	461	121.8 ± 4.8	126.1 ± 5.0	**<0.0001**
		11.2 (10.2, 12.0)	11.3 (9.9, 12.5)			10.1 (9.6, 10.8)	9.3 (8.3, 10.0)	
BMI (kg/m^2^)	438	15.6 ± 1.3	20.1 ± 1.8	**<0.0001**	461	15.3 ± 1.3	20.0 ± 1.6	**<0.0001**
		15.6 (14.7, 16.5)	19.7 (18.8, 21.2)			15.3 (14.4, 16.1)	19.7 (18.7, 20.7)	
BMI *z* score	438	−0.006 ± 0.01	0.018 ± 0.01	**<0.0001**	461	−0.007 ± 0.0	0.020 ± 0.0	**<0.0001**
		−0.005 (−0.01, 0.001)	0.017 (0.01, 0.02)			−0.006 (−0.01, 0.00)	0.020 (0.01, 0.02)	
Waist Circumference (cm)	425	55.2 ± 3.8	66.0 ± 5.9	**<0.0001**	456	54.1 ± 4.1	66.1 ± 4.0	**<0.0001**
		55.0 (52.5, 57.6)	66.9 (61.0, 69.0)			53.9 (51.0, 56.6)	65.7 (63.0, 69.0)	
**Blood pressure**								
Systolic Blood Pressure (mmHg)	412	102.4 ± 11.6	105.4 ± 11.1	0.0754	435	98.1 ± 11.9	104.5 ± 11.4	**0.0082**
		100.0 (95.0, 110.0)	100.0 (100.0, 112.5)			100.0 (90.0, 105.0)	100.0 (100.0, 115.0)	
Diastolic Blood Pressure (mmHg)	412	64.6 ± 9.1	67.8 ± 9.1	**0.0134**	435	62.8 ± 9.4	68.8 ± 8.7	**0.0018**
		65.0 (60.0, 70.0)	70.0 (60.0, 70.0)			60.0 (60.0, 70.0)	70.0 (60.0, 80.0)	
**Laboratory measurements ^2^**								
Aspartate aminotransferase (IU/L)	299	28.2 ± 5.3	30.6 ± 31.6	0.5666	310	27.2 ± 4.4	25.2 ± 4.9	**0.0226**
		27.5 (25.0, 31.0)	26.0 (23.0, 28.0)			27.0 (24.0, 30.0)	25.0 (21.0, 27.5)	
Alanine aminotransferase (IU/L)	299	14.7 ± 4.5	25.2 ± 54.4	**0.0337**	310	13.6 ± 2.7	17.0 ± 7.3	**0.0225**
		14.0 (12.0, 16.0)	16.0 (13.0, 20.0)			13.0 (12.0, 15.0)	15.0 (13.0, 18.5)	
Triglycerides (mg/dL)	297	62.0 ± 31.6	72.6 ± 38.6	0.0689	309	70.4 ± 33.4	96.8 ± 60.8	**0.0019**
		54.0 (40.0, 80.0)	68.0 (42.0, 90.0)			62.0 (48.0, 88.0)	85.5 (63.5, 108.0)	
Total cholesterol (mg/dL)	299	165.5 ± 28.3	179.4 ± 26.9	**0.0021**	310	170.8 ± 28.5	177.3 ± 26.9	0.3052
		160.0 (147.0, 180.0)	174.0 (160.0, 200.0)			169.0 (153.0, 188.0)	178.5 (155.5, 188.5)	
HDL cholesterol (mg/dL)	297	56.3 ± 10.9	53.6 ± 10.8	0.1283	309	54.5 ± 11.3	49.9 ± 10.9	**0.0365**
		56.0 (48.0, 64.0)	52.0 (47.0, 64.0)			54.0 (46.0, 60.0)	46.0 (43.0, 58.5)	
LDL cholesterol (mg/dL)	297	96.9 ± 25.1	111.3 ± 25.3	**0.0005**	309	102.2 ± 25.3	108.0 ± 24.8	0.3658
		92.2 (79.6, 110.0)	108.2 (93.0, 134.0)			102.2 (87.0, 117.6)	112.3 (95.4, 119.6)	
Glucose (mg/dL)	299	82.1 ± 10.6	83.9 ± 6.9	0.0909	310	80.2 ± 8.0	82.5 ± 7.1	0.1630
		82.5 (77.0, 87.0)	86.0 (78.0, 89.0)			80.0 (75.0, 85.0)	83.5 (77.0, 86.0)	
**Insulin resistance index ^2^**								
TyG index	297	7.7 ± 0.5	7.9 ± 0.5	**0.0453**	309	7.8 ± 0.4	8.2 ± 0.5	**0.0010**
		7.7 (7.4, 8.1)	8.0 (7.5, 8.2)			7.8 (7.5, 8.2)	8.2 (7.8, 8.5)	

Abbreviations: BMI, body mass index; TyG, triglyceride–glucose. ^1^ Including junior college. ^2^ Tested after logarithmic transformation. Data are presented as *n* (%) for categorical variables, and as mean ± SD and median (Q1, Q3) for continuous variables. *p* values were calculated via the chi-square test or Student’s *t* test, as appropriate. Bold *p* values indicate *p* < 0.05.

**Table 2:** Comparison of cardiometabolic risk factors at 15 years of age by BMI pattern.

	Boys				Girls			
	*n*	Normal Weight Maintenance group	Overweight Maintenance Group	*p*	*n*	Normal Weight Maintenance Group	Overweight Maintenance Group	*p*
*n* ** (%)**	287	240 (83.6)	47 (16.4)		288	274 (95.1)	14 (4.9)	
**Age (years)**	287	15.2 ± 0.4	15.1 ± 0.4	0.6667	288	15.1 ± 0.4	15.1 ± 0.6	0.5285
		15.2 (14.9, 15.4)	15.2 (14.8, 15.4)			15.1 (14.7, 15.4)	14.9 (14.8, 15.7)	
**Anthropometric measurements**								
Height (cm)	287	170.9 ± 5.8	171.0 ± 5.9	0.9180	288	160.3 ± 4.8	161.5 ± 4.9	0.3426
		171.0 (167.3, 174.5)	172.1 (167.3, 174.1)			160.4 (157.2, 163.0)	158.8 (158.1, 164.6)	
BMI (kg/m^2^)	287	20.2 ± 2.6	27.1 ± 3.5	**<0.0001**	288	20.3 ± 2.1	26.0 ± 3.4	**<0.0001**
		19.6 (18.5, 21.8)	27.2 (24.4, 28.9)			20.2 (18.8, 21.7)	25.9 (24.6, 26.7)	
BMI *z* score	287	−0.007 ± 0.02	0.031 ± 0.02	**<0.0001**	288	−0.003 ± 0.01	0.027 ± 0.02	**<0.0001**
		−0.010 (−0.02, 0.003)	0.032 (0.02, 0.04)			−0.003 (−0.01, 0.01)	0.027 (0.02, 0.03)	
Waist Circumference (cm)	287	71.5 ± 6.6	87.6 ± 10.4	**<0.0001**	288	68.0 ± 5.1	77.8 ± 7.5	**0.0003**
		70.0 (66.7, 75.4)	88.3 (78.6, 95.5)			67.5 (64.8, 71.0)	76.3 (71.5, 80.5)	
Body fat percentage (%)	287	15.1 ± 5.9	29.2 ± 8.2	**<0.0001**	288	30.2 ± 4.5	37.9 ± 4.3	**<0.0001**
		14.1 (10.6, 18.1)	29.4 (23.6, 34.6)			30.4 (27.3, 32.9)	38.9 (33.7, 40.6)	
Fat-free mass (kg)	287	49.9 ± 5.1	55.4 ± 5.1	**<0.0001**	288	36.2 ± 3.0	42.0 ± 5.1	**0.0010**
		50.0 (46.0, 53.4)	55.3 (51.3, 58.7)			36.1 (34.1, 38.2)	41.9 (36.7, 44.9)	
Trunk fat mass (kg)	150	4.4 ± 2.7	11.1 ± 5.2	**<0.0001**	161	8.1 ± 2.3	14.4 ± 3.6	**0.0008**
		3.8 (2.4, 5.8)	10.3 (6.5, 15.4)			8.0 (6.8, 9.4)	13.7 (12.6, 15.5)	
**Blood pressure**								
Systolic Blood Pressure (mmHg)	218	112.4 ± 10.8	121.3 ± 13.0	**0.0001**	212	103.4 ± 12.1	115.6 ± 15.1	**0.0039**
		110.0 (105.0, 120.0)	120.0 (110.0, 130.0)			100.0 (90.0, 110.0)	120.0 (110.0, 120.0)	
Diastolic Blood Pressure (mmHg)	218	68.5 ± 8.2	74.1 ± 8.6	**0.0009**	212	65.3 ± 8.8	71.1 ± 11.7	0.0562
		70.00 (60.0, 70.0)	70.00 (70.0, 80.0)			60.00 (60.0, 70.0)	70.00 (60.0, 70.0)	
**Laboratory measurements ^1^**								
Aspartate aminotransferase (IU/L)	135	19.9 ± 6.5	26.8 ± 12.5	**0.0203**	143	16.9 ± 3.6	16.9 ± 3.3	0.9523
		19.0 (16.0, 21.0)	22.5 (19.0, 32.0)			16.0 (14.0, 19.0)	16.0 (15.0, 17.0)	
Alanine aminotransferase (IU/L)	135	13.6 ± 6.0	34.7 ± 28.7	**0.0005**	143	10.7 ± 3.6	15.1 ± 8.2	0.1613
		12.0 (10.0, 15.0)	27.0 (14.0, 37.0)			10.0 (8.0, 12.0)	15.0 (9.0, 17.0)	
Triglycerides (mg/dL)	135	69.5 ± 36.7	120.7 ± 109.7	**0.0372**	143	81.5 ± 39.7	77.4 ± 20.9	0.8831
		62.0 (47.0, 87.0)	77.0 (51.0, 139.0)			74.5 (55.0, 100.5)	75.0 (64.0, 84.0)	
Total cholesterol (mg/dL)	135	149.8 ± 24.8	174.9 ± 36.3	**0.0007**	143	170.8 ± 29.6	180.4 ± 29.8	0.3880
		149.0 (132.0, 162.0)	165.0 (151.0, 192.0)			170.0 (151.5, 192.0)	180.0 (157.0, 198.0)	
HDL cholesterol (mg/dL)	135	56.9 ± 10.1	48.7 ± 10.9	**0.0008**	143	59.9 ± 10.7	55.6 ± 11.1	0.2597
		56.0 (49.0, 64.0)	47.0 (40.0, 54.0)			60.0 (51.0, 67.0)	60.0 (46.0, 62.0)	
LDL cholesterol (mg/dL)	135	79.0 ± 22.5	102.1 ± 35.7	**0.0012**	143	94.6 ± 25.5	109.4 ± 26.1	0.1396
		77.6 (63.4, 91.4)	94.3 (77.8, 122.8)			91.7 (76.3, 108.7)	104.4 (98.6, 124.0)	
Insulin (ng/mL)	135	12.1 ± 9.0	28.0 ± 23.1	**0.0032**	143	14.3 ± 10.8	17.5 ± 6.1	0.1268
		10.0 (7.4, 13.6)	24.0 (10.5, 36.4)			11.4 (8.5, 15.6)	14.8 (14.1, 20.5)	
Glucose (mg/dL)	135	92.4 ± 9.0	97.1 ± 6.8	**0.0370**	143	92.1 ± 8.4	97.4 ± 8.6	0.1294
		94.0 (88.0, 97.0)	95.5 (90.0, 104.0)			93.0 (87.5, 97.0)	100.0 (95.0, 102.0)	
**Insulin resistance ind ices ^1^**								
HOMA-IR index	135	2.8 ± 2.2	6.9 ± 6.1	**0.0026**	143	3.3 ± 2.5	4.1 ± 1.0	0.0050
		2.3 (1.6, 3.1)	5.8 (2.4, 9.3)			2.6 (1.9, 3.7)	3.8 (3.5, 5.2)	
TyG index	135	8.0 ± 0.5	8.4 ± 0.7	**0.0210**	143	8.1 ± 0.5	8.2 ± 0.3	0.6098
		8.0 (7.6, 8.3)	8.3 (7.8, 8.9)			8.1 (7.8, 8.4)	8.2 (8.1, 8.4)	

Abbreviations: BMI, body mass index; HOMA-IR, homeostasis model assessment of insulin resistance; TyG, triglyceride–glucose. ^1^ Tested after logarithmic transformation. Data are presented as *n* (%) for categorical variables, and as mean ± SD and median (Q1, Q3) for continuous variables. *p* values were calculated using Student’s *t* test. Bold *p* values indicate *p* < 0.05.

**Table 3:** Comparison of cardiometabolic risk factors at 18 years of age by BMI pattern.

	Boys				Girls			
	*n*	Normal Weight Maintenance Group	Overweight Maintenance Group	*p*	*n*	Normal Weight Maintenance Group	Overweight Maintenance Group	*p*
*n* ** (%)**	108	94 (87.0)	14 (13.0)		92	90 (97.8)	2 (2.2)	
**Age (years)**	108	18.3 ± 0.3	18.3 ± 0.3	0.9757	92	18.3 ± 0.3	18.6 ± 0.1	0.2205
		18.3 (18.1, 18.5)	18.3 (18.1, 18.5)			18.3 (18.0, 18.6)	–	
**Anthropometric measurements**								
Height (cm)	108	175.0 ± 5.2	170.8 ± 6.2	**0.0075**	92	161.5 ± 4.9	162.3 ± 8.2	0.8103
		174.9 (172.1, 177.9)	169.7 (166.2, 175.8)			161.6 (157.9, 164.5)	–	
BMI (kg/m^2^)	108	22.4 ± 3.5	28.4 ± 4.8	**<0.0001**	92	21.5 ± 3.0	33.9 ± 5.4	**<0.0001**
		21.8 (20.0, 24.5)	27.0 (24.8, 34.0)			21.0 (19.6, 23.0)	–	
BMI *z* score	108	−0.001 ± 0.02	0.028 ± 0.02	**<0.0001**	92	0.001 ± 0.02	0.046 ± 0.01	**<0.0001**
		−0.003 (−0.01, 0.011)	0.022 (0.01, 0.05)			0.0003 (−0.01, 0.01)	–	
Waist Circumference (cm)	108	78.1 ± 8.9	90.7 ± 11.4	**<0.0001**	92	70.9 ± 9.0	96.4 ± 3.3	**0.0001**
		78.0 (72.0, 84.6)	89.6 (80.0, 100.0)			71.0 (66.4, 75.0)	–	
Body fat percentage (%)	107	20.5 ± 7.0	31.2 ± 9.0	**<0.0001**	92	32.8 ± 5.4	48.7 ± 0.8	**<0.0001**
		20.1 (15.9, 25.4)	27.0 (25.9, 38.5)			33.2 (30.0, 36.9)	–	
Fat-free mass (kg)	107	53.8 ± 5.3	55.8 ± 4.5	0.1635	92	37.3 ± 3.6	46.3 ± 11.2	0.4608
		53.3 (50.1, 57.3)	55.5 (52.4, 56.9)			36.8 (34.8, 39.0)	–	
Trunk fat mass (kg)	107	8.4 ± 4.4	15.1 ± 7.2	**0.0040**	91	10.3 ± 3.0	24.6 ± 4.5	**<0.0001**
		7.4 (5.5, 10.8)	12.5 (10.2, 22.4)			9.8 (8.1, 12.0)	–	
**Blood pressure**								
Systolic Blood Pressure (mmHg)	79	116.3 ± 8.8	117.1 ± 7.0	0.7557	82	101.4 ± 7.3	126.0 ± 5.7	**<0.0001**
		118.0 (110.0, 120.0)	120.0 (116.0, 120.0)			100.0 (96.0, 109.0)	–	
Diastolic Blood Pressure (mmHg)	79	73.7 ± 8.9	73.8 ± 7.5	0.9595	82	63.5 ± 4.4	84.0 ± 8.5	**<0.0001**
		70.00 (70.0, 80.0)	76.00 (70.0, 80.0)			62.00 (60.0, 65.0)	–	
**Laboratory measurements ^1^**								
Aspartate aminotransferase (IU/L)	63	26.0 ± 25.5	49.1 ± 43.6	**0.0010**	65	19.8 ± 7.9	36.0 ± 21.2	**0.0182**
		20.5 (17.0, 24.0)	37.0 (23.0, 50.0)			17.0 (15.0, 21.0)	–	
Alanine aminotransferase (IU/L)	63	23.1 ± 21.5	64.6 ± 44.0	**<0.0001**	64	16.6 ± 13.3	67.5 ± 55.9	**0.0010**
		17.0 (13.0, 24.5)	81.0 (20.0, 95.0)			12.0 (9.0, 17.0)	–	
Triglycerides (mg/dL)	63	96.2 ± 56.5	140.7 ± 123.9	0.1104	65	76.9 ± 33.5	77.5 ± 36.1	0.9046
		82.5 (57.0, 122.0)	109.0 (73.0, 171.0)			64.0 (55.0, 102.0)	–	
Total cholesterol (mg/dL)	63	163.8 ± 29.3	182.7 ± 44.0	0.1194	65	174.3 ± 33.3	183.5 ± 13.4	0.6195
		156.0 (145.0, 180.0)	173.0 (144.0, 222.0)			174.0 (144.0, 198.0)	–	
HDL cholesterol (mg/dL)	63	55.6 ± 10.6	50.1 ± 8.3	0.1290	65	61.8 ± 11.7	55.0 ± 1.4	0.4651
		57.0 (46.0, 62.0)	49.0 (42.0, 54.0)			60.0 (54.0, 70.0)	–	
LDL cholesterol (mg/dL)	63	89.0 ± 28.0	104.5 ± 30.3	0.1672	65	97.1 ± 29.5	113.0 ± 7.6	0.3773
		80.7 (72.5, 107.4)	102.4 (72.0, 137.6)			97.8 (71.6, 116.0)	–	
Insulin (ng/mL)	63	16.3 ± 14.5	29.6 ± 28.6	0.0661	65	13.9 ± 19.0	24.8 ± 0.4	**<0.0001**
		11.1 (7.5, 18.2)	19.3 (10.4, 41.9)			10.0 (6.4, 14.1)	–	
Glucose (mg/dL)	63	92.3 ± 10.1	91.6 ± 5.7	0.9237	65	89.9 ± 8.0	106.5 ± 10.6	**0.0105**
		91.0 (87.0, 101.0)	91.0 (86.0, 97.0)			90.0 (86.0, 95.0)	–	
**Insulin resistance ind ices ^1^**								
HOMA-IR index	63	3.9 ± 3.7	6.8 ± 7.0	0.0833	65	3.3 ± 5.2	6.5 ± 0.7	0.0528
		2.6 (1.7, 4.2)	4.8 (2.2, 8.9)			2.1 (1.4, 3.1)	–	
TyG index	63	8.3 ± 0.6	8.5 ± 0.7	0.1410	65	8.1 ± 0.4	8.3 ± 0.4	0.4958
		8.2 (7.9, 8.6)	8.5 (8.0, 8.9)			8.0 (7.8, 8.4)	–	

Abbreviations: BMI, body mass index; HOMA-IR, homeostasis model assessment of insulin resistance; TyG, triglyceride–glucose. ^1^ Tested after logarithmic transformation. Data are presented as *n* (%) for categorical variables, and as mean ± SD and median (Q1, Q3) for continuous variables. –, not available due to small sample size. *p* values were calculated by Student’s *t* test. Bold *p* values indicate *p* < 0.05.

**Table 4:** Associations between BMI patterns and cardiometabolic risk factors at ages 15 and 18 in boys.

		Unadjusted		Adjusted ^1^	
	Normal Weight Maintenance Group	Overweight Maintenance Group	*p*	Overweight Maintenance Group	*p*
	*β* (95% CI)	*β* (95% CI)		*β* (95% CI)	
**at 15 years**					
BMI (kg/m^2^)	Reference	0.290 (0.247, 0.333)	**<0.0001**	0.254 (0.209, 0.299)	**<0.0001**
Waist Circumference (cm)	Reference	0.200 (0.167, 0.233)	**<0.0001**	0.172 (0.138, 0.207)	**<0.0001**
Body fat percentage (%)	Reference	0.694 (0.560, 0.828)	**<0.0001**	0.605 (0.465, 0.746)	**<0.0001**
Fat-free mass (kg)	Reference	0.105 (0.070, 0.140)	**<0.0001**	0.083 (0.047, 0.120)	**<0.0001**
Trunk fat mass (kg)	Reference	0.992 (0.696, 1.288)	**<0.0001**	0.866 (0.551, 1.181)	**<0.0001**
Systolic Blood Pressure (mmHg)	Reference	0.075 (0.036, 0.114)	**0.0002**	0.061 (0.019, 0.102)	**0.0046**
Diastolic Blood Pressure (mmHg)	Reference	0.080 (0.033, 0.127)	**0.0010**	0.061 (0.014, 0.109)	**0.0119**
Aspartate aminotransferase (IU/L)	Reference	0.251 (0.111, 0.390)	**0.0005**	0.294 (0.140, 0.448)	**0.0002**
Alanine aminotransferase (IU/L)	Reference	0.737 (0.525, 0.948)	**<0.0001**	0.761 (0.528, 0.994)	**<0.0001**
Triglycerides (mg/dL)	Reference	0.392 (0.132, 0.652)	**0.0034**	0.293 (0.002, 0.584)	**0.0486**
HDL cholesterol (mg/dL)	Reference	−0.163 (−0.257, −0.069)	**0.0008**	−0.160 (−0.264, −0.057)	**0.0028**
HOMA-IR index	Reference	0.751 (0.442, 1.061)	**<0.0001**	0.691 (0.343, 1.039)	**0.0001**
**at 18 years**					
BMI (kg/m^2^)	Reference	0.235 (0.148, 0.322)	**<0.0001**	0.207 (0.119, 0.294)	**<0.0001**
Waist Circumference (cm)	Reference	0.149 (0.083, 0.215)	**<0.0001**	0.136 (0.068, 0.203)	**0.0001**
Body fat percentage (%)	Reference	0.442 (0.245, 0.639)	**<0.0001**	0.407 (0.207, 0.607)	**0.0001**
Fat-free mass (kg)	Reference	0.040 (−0.014, 0.094)	0.1490	0.030 (−0.025, 0.084)	0.2860
Trunk fat mass (kg)	Reference	0.615 (0.326, 0.904)	**<0.0001**	0.566 (0.274, 0.858)	**0.0002**
Systolic Blood Pressure (mmHg)	Reference	0.008 (−0.036, 0.052)	0.7197	0.015 (−0.033, 0.062)	0.5453
Diastolic Blood Pressure (mmHg)	Reference	0.003 (−0.063, 0.069)	0.9263	−0.006 (−0.076, 0.063)	0.8536
Aspartate aminotransferase (IU/L)	Reference	0.584 (0.247, 0.921)	**0.0010**	0.658 (0.296, 1.020)	**0.0006**
Alanine aminotransferase (IU/L)	Reference	0.978 (0.569, 1.386)	**<0.0001**	0.989 (0.554, 1.425)	**<0.0001**
Triglycerides (mg/dL)	Reference	0.289 (−0.068, 0.645)	0.1104	0.181 (−0.167, 0.530)	0.3015
HDL cholesterol (mg/dL)	Reference	−0.097 (−0.223, 0.029)	0.1290	−0.092 (−0.228, 0.043)	0.1763
HOMA-IR index	Reference	0.478 (−0.065, 1.021)	0.0833	0.371 (−0.218, 0.960)	0.2120

Abbreviations: BMI, body mass index; HOMA-IR, homeostasis model assessment of insulin resistance. ^1^ Adjusted for baseline age, maternal education, maternal BMI status, paternal BMI status, and daily sedentary time. Bold *p* values indicate *p* < 0.05. Tested after logarithmic transformation.

## Data Availability

The data presented in this study are available upon request from the corresponding author. The data are not publicly available due to privacy or ethical restrictions.

## Supplementary Materials

The following supporting information can be downloaded at: https://www.mdpi.com/article/10.3390/children12070821/s1, Table S1: Comparison of baseline characteristics between participants retained and lost to follow-up at age 15; Table S2: Comparison of baseline characteristics between participants retained and lost to follow-up at age 18.
