# Supplementary material for: Sex-Specific Associations of Childhood BMI Patterns with Cardiometabolic Risk: An 11-Year Korean Longitudinal Study
Source: Children (Basel). 2025 Jun 21;12(7):821. doi: 10.3390/children12070821 (PMC12293192; doi:10.3390/children12070821)
Supplement: Supplementary file 1 [file children-12-00821-s001.zip › children-3643987-supplementary.pdf]

Supplementary Table S1. Comparison of baseline characteristics between participants retained and lost to follow-up at age 15

	Boys				Girls			
	**N**	**Retained**	**Lost to follow-up**	***P***	**N**	**Retained**	**Lost to follow-up**	***P***
N **(%)**	438	287 (65.5)	151 (34.5)		461	288 (62.5)	173 (37.5)	
**Age (years)**	438	7.1 ± 0.4	7.2 ± 0.4	0.0264	461	7.0 ± 0.4	7.2 ± 0.3	<0.0001
**BMI pattern, n (%)**	438			0.9266	461			0.2401
Normal weight maintenance group		250 (87.1)	132 (87.4)			274 (95.1)	160 (92.5)	
Overweight maintenance group		37 (12.9)	19 (12.6)			14 (4.9)	13 (7.5)	
**Maternal education, n (%)**	438			0.4213	461			0.0565
High school or lower		56 (19.5)	31 (20.5)			60 (20.8)	45 (26.0)	
University[1] or higher		162 (56.5)	76 (50.3)			163 (56.6)	78 (45.1)	
Missing		69 (24.0)	44 (29.2)			65 (22.6)	50 (28.9)	
**Maternal employment status, n (%)**	438			0.9874	461			**0.0122**
Not working		108 (37.6)	57 (37.8)			125 (43.4)	84 (48.6)	
Working		97 (33.8)	50 (33.1)			96 (33.3)	36 (20.8)	
Missing		82 (28.6)	44 (29.1)			67 (23.3)	53 (30.6)	
**Maternal BMI status, n (%)**	438			0.0917	461			0.0990
Normal weight		163 (56.8)	90 (59.6)			171 (59.4)	85 (49.1)	
Overweight		48 (16.7)	14 (9.3)			44 (15.3)	32 (18.5)	
Missing		76 (26.5)	47 (31.1)			73 (25.3)	56 (32.4)	
**Paternal BMI status, n (%)**	438			0.3342	461			0.0891
Normal weight		98 (33.4)	42 (27.8)			84 (29.2)	57 (32.9)	
Overweight		113 (39.4)	59 (39.1)			131 (45.5)	61 (35.3)	
Missing		78 (27.2)	50 (33.1)			73 (25.3)	55 (31.8)	
**Sedentary time, n (%)**	438			0.7064	461			**0.0366**
< 1hour		71 (24.7)	32 (21.2)			59 (20.5)	30 (17.3)	
>= 1 hour		86 (30.0)	47 (31.1)			111 (38.5)	51 (29.5)	
Missing		130 (45.3)	72 (47.7)			118 (41.0)	92 (53.2)	
**Walking time, n (%)**	438			0.0986	461			0.0092
< 1 hour		149 (52.0)	87 (57.6)			153 (53.1)	103 (59.5)	
> =1 hour		69 (24.0)	23 (15.2)			80 (27.8)	27 (15.6)	
Missing		69 (24.0)	41 (27.2)			55 (19.1)	43 (24.9)	
**Anthropometric measurements**								
Height (cm)	438	122.8 ± 5.4	124.2 ± 4.9	**0.0078**	461	121.3 ± 5.1	123.4 ± 4.3	**<0.0001**
BMI (kg/m^2)	438	16.3 ± 2.0	16.1 ± 2.0	0.5607	461	15.6 ± 1.6	15.6 ± 1.9	0.9076
BMI z-score	438	-0.002 ± 0.01	-0.004 ± 0.01	0.394	461	-0.005 ± 0.0	-0.006 ± 0.0	0.5207
Waist Circumference (cm)	425	56.4 ± 5.5	57.1 ± 5.5	0.1918	456	54.3 ± 4.7	55.7 ± 5.3	**0.0035**
**Blood pressure**								
Systolic Blood Pressure (mmHg)	412	102.4 ± 11.2	103.6 ± 12.3	0.3413	435	98.6 ± 11.6	98.4 ± 12.5	0.8561
Diastolic Blood Pressure (mmHg)	412	64.6 ± 9.0	65.8 ± 9.5	0.2082	435	62.9 ± 9.2	63.8 ± 10.0	0.3244

Abbreviations: BMI, body mass index; TyG, triglyceride-glucose.

[1] Including junior college

Data are presented as n (%) or mean ± SD.

P values were calculated via the chi-square test or Student's t test, as appropriate.

Bold values indicate P < 0.05.

Supplementary Table S2. Comparison of baseline characteristics between participants retained and lost to follow-up at age 18

	Boys				Girls			
	N	Retained	Lost to follow-up	*P*	N	Retained	Lost to follow-up	*P*
***N* (%)**	438	108 (24.7)	330 (75.3)		461	92 (20.0)	369 (80.0)	
**Age (years)**	438	7.0 ± 0.3	7.2 ± 0.4	**<0.0001**	461	7.0 ± 0.4	7.1 ± 0.4	**<0.0001**
**BMI pattern, n (%)**	438			0.9492	461			0.0927
Normal weight maintenance group		94 (87.0)	288 (87.3)			90 (97.8)	334 (93.2)	
Overweight maintenance group		14 (13.0)	42 (12.7)			2 (2.2)	25 (6.8)	
**Maternal education, n (%)**	438			0.5642	461			0.1005
High school or lower		21 (19.5)	66 (20.0)			23 (25.0)	82 (22.2)	
University[1] or higher		63 (58.3)	175 (53.0)			54 (58.7)	187 (50.7)	
Missing		24 (22.2)	89 (27.0)			15 (16.3)	100 (27.1)	
**Maternal employment status, n (%)**	438			0.9526	461			0.0997
Not working		41 (38.0)	124 (37.6)			48 (52.2)	161 (43.6)	
Working		35 (32.4)	112 (33.9)			28 (30.4)	104 (28.2)	
Missing		32 (29.6)	94 (28.5)			16 (17.4)	104 (28.2)	
**Maternal BMI status, n (%)**	438			0.9744	461			**0.0220**
Normal weight		62 (57.4)	191 (57.9)			62 (67.4)	194 (52.6)	
Overweight		16 (14.8)	46 (13.9)			14 (15.2)	62 (16.8)	
Missing		30 (27.8)	93 (28.2)			16 (17.4)	113 (30.6)	
**Paternal BMI status, n (%)**	438			0.1558	461			**0.0293**
Normal weight		42 (38.9)	96 (29.1)			37 (40.2)	104 (28.2)	
Overweight		39 (36.1)	133 (40.3)			38 (41.3)	154 (41.7)	
Missing		27 (25.0)	101 (30.6)			17 (18.5)	111 (30.1)	
**Sedentary time, n (%)**	438			0.2306	461			**0.0006**
< 1hour		31 (28.7)	72 (21.8)			18 (19.6)	71 (19.2)	
>= 1 hour		34 (31.5)	99 (30.0)			47 (51.1)	115 (31.2)	
Missing		43 (39.8)	159 (48.2)			27 (29.3)	183 (49.6)	
**Walking time, n (%)**	438			0.5433	461			**0.0014**
< 1 hour		60 (55.6)	176 (53.3)			58 (63.0)	198 (53.7)	
> =1 hour		25 (23.1)	67 (20.3)			27 (29.4)	80 (21.7)	
Missing		23 (21.3)	87 (26.4)			7 (7.6)	91 (24.6)	
**Anthropometric measurements**								
Height (cm)	438	121.9 ± 4.9	123.8 ± 5.3	**0.0014**	461	120.3 ± 5.2	122.5 ± 4.8	**0.0001**
BMI (kg/m^2)	438	16.3 ± 2.2	16.2 ± 2.0	0.5665	461	15.3 ± 1.7	15.6 ± 1.7	0.1045
BMI z-score	438	-0.002 ± 0.01	-0.003 ± 0.01	0.3621	461	-0.007 ± 0.0	-0.005 ± 0.0	0.1935
Waist Circumference (cm)	425	56.0 ± 5.8	56.9 ± 5.4	0.1628	456	53.0 ± 4.5	55.3 ± 5.0	**<0.0001**
**Blood pressure**								
Systolic Blood Pressure (mmHg)	412	101.6 ± 11.0	103.3 ± 11.7	0.2031	435	96.2 ± 11.7	99.1 ± 11.9	**0.0373**
Diastolic Blood Pressure (mmHg)	412	63.2 ± 9.6	65.7 ± 9.0	0.0154	435	60.5 ± 9.7	63.9 ± 9.3	**0.0019**

Abbreviations: BMI, body mass index; TyG, triglyceride-glucose.

[1] Including junior college

Data are presented as n (%) or mean ± SD.

P values were calculated via the chi-square test or Student's t test, as appropriate.

Bold values indicate P < 0.05.
